# Mixed-Thickness Tunnel Access (MiTT) through a Linear Vertical Mucosal Incision for a Minimally Invasive Approach for Root Coverage Procedures in Anterior and Posterior Sites: Technical Description and Case Series with 1-Year Follow-Up

**DOI:** 10.3390/dj11100235

**Published:** 2023-10-07

**Authors:** Tiago Marques, Nuno Bernardo Malta dos Santos, Manuel Sousa, Juliana Campos Hasse Fernandes, Gustavo Vicentis Oliveira Fernandes

**Affiliations:** 1 Faculty of Dental Medicine, Universidade Católica Portuguesa, 3504-505 Viseu, Portugal; tmmarques@ucp.pt (T.M.);; 2 Centre for Interdisciplinary Research in Health (CIIS), Universidade Católica Portuguesa, 3504-505 Viseu, Portugal; 3 Periodontics and Oral Medicine Department, University of Michigan School of Dentistry, Ann Arbor, MI 48109, USA

**Keywords:** clinical study, gingival recession, periodontics, surgical procedures

## Abstract

Purpose: The goal of this article was to introduce a new root coverage (RC) technique, the mixed-thickness tunnel access (MiTT) technique, which approaches a full-split design and intends to augment soft tissues coronal to the gingival margin. It was shown step-by-step, and the results were presented in a case series. Methods: Healthy individuals (non-diabetics) and non-smokers with gingival recession (GR) type 1 or 2 (RT1 or RT2) were included. After evaluation, prophylaxis was performed 14 days before the surgical procedure. During the surgical appointment, one or two vertical incision(s) on the mucosa (around 1–2 mm apical to the MGJ), lateral to the papilla base, was/were performed after anesthesia. Initially, there was a partial incision to detach the mucosa of the muscles (split design). It was permitted (but not mandatory) to perform intrasulcular incisions. Through the vertical incision, internally, subperiosteal access from the MGJ toward the gingival margin (coronally) was performed to create a full-thickness tunnel. Then, communication from the vertical incision with the gingival sulcus and the papilla base occurred, keeping the papilla tip intact. A connective tissue graft was harvested and inserted through the linear incision or intrasulcularly. There were interrupted sutures. An adjunctive material may be applied (e.g., Endogain). The root coverage was measured using a periodontal probe and considered fully covered when the gingival margin was 1 mm coronal to the cementum–enamel junction (CEJ). Results: Nine healthy individuals (seven females and two males) aged 19 and 43 were enrolled. They were treated following the MiTT steps. Four cases had a single GR; two patients had two teeth involved; and three others had three or four GR. There were seven cases of RT1 and two RT2. All RT1 cases achieved 100% RC, while the mean RC obtained for RT2 was around 80%. Conclusion: The MiTT technique can be considered a more straightforward approach for minimally invasive surgical techniques, which is a feasible option to treat RC with a high success rate, predictability, and esthetic preservation. Therefore, there is a technical sensitivity to performing the full-split design procedure.

## 1. Introduction

Pursuing a more predictable surgical procedure to treat gingival recessions has led to technological advances in recent years. Hence, many therapeutic options are available, including soft tissue tunneling [1]. This is a widely accepted procedure even though it is a highly sensitive and blind technique. It may cause sulcular epithelium and soft tissue trauma, resulting in less-than-acceptable outcomes. Several tunneling methods were proposed to preserve esthetics, maintain papillary integrity, and prevent relapse and scarring resulting from vertical releasing incisions in keratinized tissue [2,3]. The first technique (the “Envelope” technique) [4], published in 1985, is a procedure without coronal displacement of the mucogingival junction. A connective tissue graft (CTG) is placed over the recession. Therefore, the exposed graft has a greater risk of necrosis for teeth with a denuded root length above 3 mm. Then, accesses through new areas to create the tunnel access are available, typically in the alveolar mucosa (apical to the mucogingival junction).

The vestibular incision subperiosteal tunnel access (VISTA) technique [5] was introduced in this context. It presented a modification for the existing modified envelope [6,7] and tunnel technique [2]. It uses new access through the frenum and creates the subperiosteal tunnel instead of the previous supraperiosteal approach. Thus, a total soft tissue elevation (epithelium, connective tissue, and periosteum) is performed. The tissue is gently raised in the interproximal area and the base of each papilla without any superficial incisions. This tunnel communicates with the intrasulcular area and must be sufficiently elevated (beyond the mucogingival junction and through the gingival sulcus of the respective tooth) to reach a low-tension coronal repositioning of the gingiva. In addition, the original technique also includes a membrane complex (beta-tricalcium phosphate (β-TCP) hydrated with rhPDGF-BB).

Another technique named the Pinhole^®^ surgical technique (PST) was also reported, which elevates the periosteum (full-thickness flap) [8]. This technique makes a hole (around 2–3 mm using specific instruments) in the base of the buccal mucosa, apical to the recession in case of a single defect, or, in case of multiple recessions, in the interradicular area of two adjacent defect sites. Similar to VISTA, an intrasulcular incision is made, keeping the tip of the interdental papilla intact. A specific tunneling instrument (transmucosal periosteal elevator) is inserted through the pinhole and used for blunt dissection. The flap is coronally and horizontally extended. The original technique uses collagen stripes placed through the pinhole beneath the tunnel for stabilization.

A similar procedure was preconized by Tuttle et al. (2018) [9], which substituted the connective tissue graft (CTG) for advanced-platelet rich fibrin (A-PRF) (centrifuged to 1300 rpm/8 min) and injectable-PRF (i-PRF) (700 rpm/3 min). It was named the gum drop technique (GDT), a minimally invasive and biologically enhanced soft-tissue procedure, which places holes (around four for the entire arch) in the gingiva apical to the mucogingival junction (MGJ) using a small soft-tissue piercing instrument. Appropriate instruments are used to create a full-thickness tunnel (subperiosteal access). The tissue is detached and advanced coronally, except for the papillae, which are spared and not detached. The flap is then sutured preferentially 2 mm coronal to the CEJ. As reported in an umbrella review developed by Fernandes et al. (2021) [10], CTG is yet the gold standard biomaterial for root coverage procedures. However, PRF may be considered a feasible substitute for treating GR defects. Three studies with a 12-month follow-up on treated recession type-1 (RT1) or Miller classification 1 and 2 patients [11,12,13] reported favorable results. Jankovic et al. [11] and Tunalι et al. [12] reported similar results between control and test groups (PRF) for the mean root coverage (MRC), respectively, 70.5% ± 11.76% and 72.1% ± 9.55% [11], and 77.36% vs. 76.63% for MRC [12]. In contrast, Kuka et al. [13] reported a higher MRC in a test group 88.36% ± 15.45% compared with 74.63% ± 8.05% in a control group. Therefore, limitations in the use of PRF can be considered the degradation time of the PRF during the healing period, questionable utilization in deep recessions, and short width of keratinized tissue.

In an attempt to evolve the technique, the periosteum was kept intact, and a supraperiosteal approach was developed [14]. Then, this tunnel access modification became a more predictable option. The idea was that a supraperiosteal tunnel approach would permit double vascular surfaces for revascularization of the graft (from the buccal flap/interdental papillae and the underlying periosteum), which is extremely important for the healing process as it guarantees nutrient supply and revascularization [15]. It would result in less graft necrosis, scarring, and capillary ingrowth, permitting optimal tissue blending. Lee et al. (2015) proposed the modified-vestibular incision supraperiosteal tunnel access (M-VISTA) technique [14], working over the principles of minimally invasive surgery. It avoids potential complications with tunneling techniques, such as VISTA incision design, supraperiosteal tunnel access instead of the original subperiosteal approach, and the graft used [5].

Nevertheless, this technique is limited because the access is only through the frenum area, where the authors proposed a “V-shaped” incision for a simultaneous frenectomy. This fact limits its application only in the anterior region. Otherwise, the difficulty found in thin phenotypes was left aside, which will present high chances of mucosal fenestration. Although M-VISTA uses a different approach, it may have an unfavorable post-operative period and is restricted to the same area. The advantages and disadvantages of the techniques abovementioned are listed in Table 1. Within this scenario, this article aimed to introduce a new method for root coverage (RC), the mixed-thickness tunnel access (MiTT) technique, approaching a full-split design, the results of which are presented as a case series. A more straightforward technique is demonstrated step-by-step, along with the details, pros, and cons. The primary outcome observed was RC, and the secondary results were pocket depth (PD) and keratinized tissue width (KTW).

## 2. Materials and Methods

This study followed the Declaration of Helsinki (2013) and was approved by the local Ethical Committee of the University (Universidade Católica Portuguesa, Viseu, Portugal). All patients received information about the technique proposed and signed an informed consent form before beginning this study. After all necessary evaluations, prophylaxis (regular cleaning, scaling root planing [SRP], and polishing) was performed 14 days before the surgical procedure.

### 2.1. Eligibility Criteria

The criteria considered for inclusion were:

1. Healthy individuals;
2. Non-smokers;
3. Non-diabetics;
4. Diagnosed with gingival recession type 1 or 2 (RT1 or RT2) [16].

The exclusion criteria were:

1. Patients with a poor standard of plaque control and demonstrating a lack of ability to maintain a good level of oral hygiene (full-mouth plaque score ≥ 20%);
2. Bleeding on probing (BoP) > 10%;
3. Questionable long-term prognosis of patient dentition;
4. Any mobility;
5. Pregnancy;
6. Severe cardiovascular disease;
7. Taking any medication that may interfere with the healing;
8. Malignancy;
9. Bleeding disorders.

### 2.2. MiTT Technique—Preparation Steps

To apply MiTT, it is necessary to analyze the following points:

1. Systemic health condition compatible with a healthy patient or with controlled disease;
2. Non-pregnant;
3. If using any medication, it must not harm healing or cause excessive bleeding;
4. Adequate blood pressure (lower than 140/90 mmHg recommended);
5. The width of the local keratinized tissue width (KTW) remnant is suggested to be at least 1 mm;
6. Whether it is a single tooth or multiple teeth with gingival recessions, evaluate the best site for the primary incision or, if necessary, more than one incision;
7. Identify the type of recession (RT) [13], which can help with the prediction of the results;
8. Verify if there is a step and visible cement–enamel junction (CEJ) [17];
9. Verify gingival thickness;
10. Verify if there is any bone or soft tissue defect close to the recession(s);
11. Periodontal diagnosis;
12. Verify if there is a rotated, tilted, or crowded tooth associated with the area of the recession
13. BoP—recommended ≤ 10%.

### 2.3. MiTT Technique—Surgical Steps

**Figure 1:**
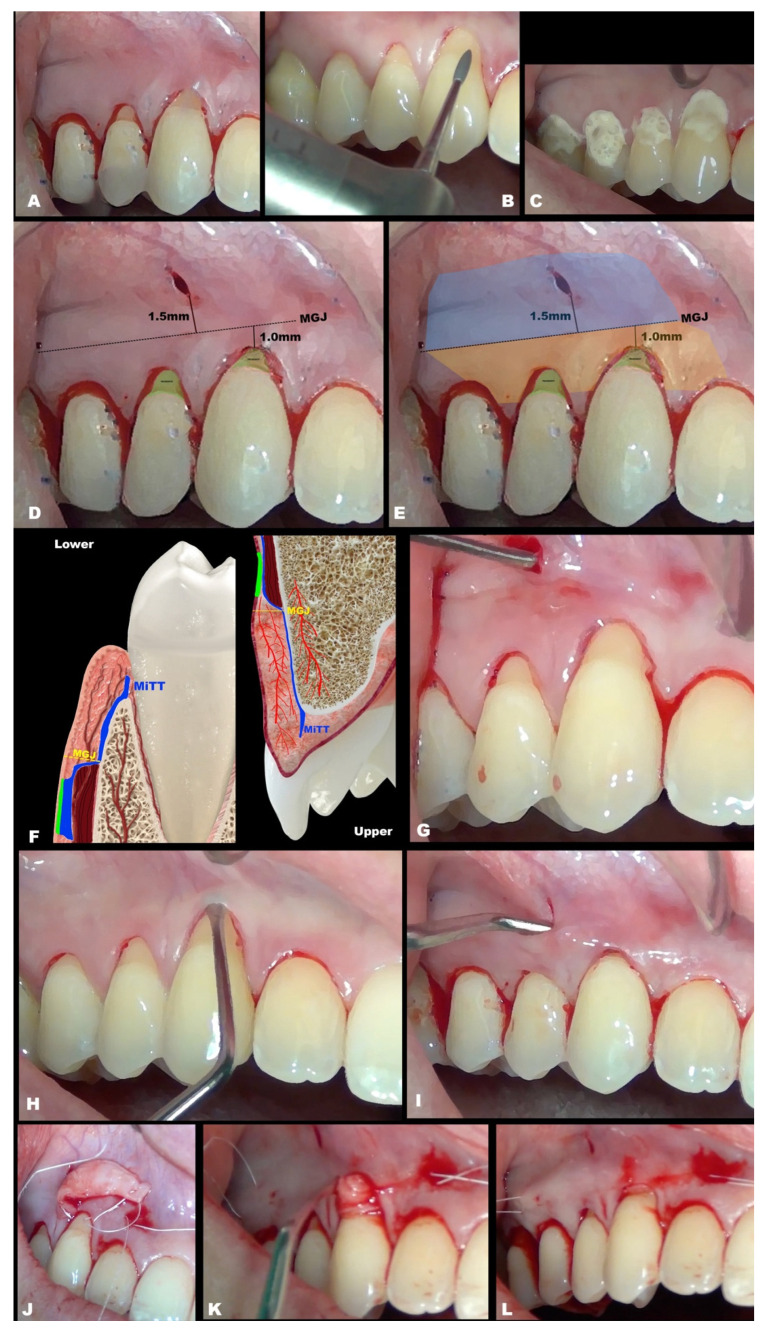
(**A**). Schematic drawing presenting two recessions (initial presentation). (**B**). Schematic drawing showing the tooth preparation with Perio Set bur. (**C**). Schematic drawing demonstrating tetracycline use for 1 min in the recessions and adjacent tissues. Rinsed with sterile saline to wash and dried using cotton pellets. Enamel matrix derivative (EMD) was applied to improve regenerative performance. (**D**). Schematic drawing showing the KTW, MGJ, and the suggestive distance from the MGJ to the vertical incision (between 1 and 2 mm). (**E**). Schematic drawing showing the first recommended area to work (apical to MGJ), in blue, and the second region localized coronal to the MGJ, in yellow. (**F**). Illustrative images showing the sagittal plane with schematic designs of the tunnel preparation with MiTT (blue line), detaching mucosa of the muscles (brown), keeping the vascularization, and showing the reduced risk of fenestration for the lower and upper tooth. (**G**). Schematic drawing showing mucosa detachment, with split and superficial approaches (see the instrument through the tissue), dividing mucosa from the muscles (deep tissue). (**H**). Schematic drawing showing intrasulcular incisions, which are not mandatory. (**I**). Schematic drawing showing full (subperiosteal) detachment of the soft tissue (instrument deeply positioned), reaching the gingival margin and base of the papilla. (**J**). Schematic drawing showing obtention of the connective tissue graft (CTG), which will be inserted in the site receptor. (**K**). Schematic drawing showing both sides of the CTG already inserted. (**L**). Schematic drawing presenting the CTG in position, using two sutures, one in the mesial and another for the distal. Then, through the sutures, the CTG will be positioned 1 mm coronal to the cement–enamel junction (CEJ) and stabilized.

The primary conduct (Figure 1A) involved previous prophylaxis (with scaling and root planing, if needed, suggested 2 weeks before the surgical procedure) was performed. Afterward, 1 min gaggling with chlorohexidine 0.12% mouthwash was performed. Then, the following were performed: composite removal at the cervical area (if existent); the surgical site was polished and adjusted with specific burs (Perio Set burs kit, recommended) (Figure 1B) to remove any contaminated cementum; include and polymerize interproximal composite (if applicable); any adjunctive treatment, if used, such as EDTA 24% for 2 min and enamel matrix derivatives (EMDs) or other materials (not mandatory) (Figure 1C); anesthesia; and delimitate the MGJ. The steps included:

(1) Vertical incision on the mucosa (around 1–2 mm apical to the MGJ), lateral to the papilla base (Figure 1D). It is mandatory not to perform this incision in the center of the papilla’s base, which might damage any vascular supply or risk damage to the papilla. In multiple recessions, it is recommended to perform two vertical incisions; and if extremely necessary, more vertical incisions can be performed, always in mucosa and lateral in the papilla’s base.
(2) Initial detachment of the mucosa from the muscles (split design, Figure 1E (blue color)–Figure 1G), apical to the MGJ with specific tunnel instruments that are commercially available, must be performed to prevent tension during coronal advancement, enrolling one adjacent tooth.
(3) It is permitted (but not mandatory) to perform intrasulcular incisions, including up to one adjacent tooth (Figure 1H), which can facilitate the procedure to connect the tunnel. Avoid causing any damage to the gingival margin.
(4) From the MGJ, subperiosteal access to raise the full-thickness tunnel is performed (Figure 1I), involving one adjacent tooth, to keep the local vascularization. The access is subperiosteal, and it is essential to act gently in this stage.
(5) Confirm the tissue detachment until the gingival sulcus area (free gingival margin) and also in the papilla’s base (Figure 1J), keeping the papilla’s tip intact.
(6) After CTG is harvested (either subepithelial or de-epithelialized), it will be inserted in the desired site through the linear incision or intrasulcularly (Figure 1K,L).
(7) The CTG will be adjusted to cover the recession (Figure 1l) and must be coronally advanced at least 1 mm coronal to the CEJ.
(8) Then, MiTT should be sutured according to the personally preferred technique. It is suggested that the suture techniques slightly pull the tunnel coronal (anchored with composite or double-crossed suture [18]). The vertical incision must be sutured with one or two single sutures. It is suggested to stabilize the soft tissues using interrupted sutures, and it may be used as adjunctive material, such as a biological glue. It is suggested that the suture be removed between 7 and 14 days.

### 2.4. Statistical Analysis

The data from the baseline and final result for pocket depth (PD), keratinized tissue width (KWT), and gingival recession height were statistically evaluated. First, the data were assessed for a normal distribution. Then, an unpaired *t*-test was applied, considering a significance level of *p* ≤ 0.05.

## 3. Results

A total of nine patients (seven females and two males), aged 19 to 43 years, needing RC procedures were initially evaluated. After the evaluation, nine cases were included that met the eligibility criteria (described below). All cases followed the technique described above, adjusting to any particularities. All patients agreed to return after 7–14 days (suture removal), 180 days, and after one year. Then, the MiTT technique was applied. The same surgeons operated and assisted patients (T.M. and N.B.M.d.S.). Registration occurred between 2018 and 2021 in the clinic at the university.

All surgical procedures were performed with a 15C blade or microblade, inserted intrasulcularly to perform the vertical incision in the mucosa, accompanied by all periodontal microsurgical kits and tunnel instruments. The measurements were performed using a periodontal probe (Hu-Friedy Color-Coded Single-End Unc Probe 1–15 1/Each, Chicago, IL, USA).

**Figure 2:**
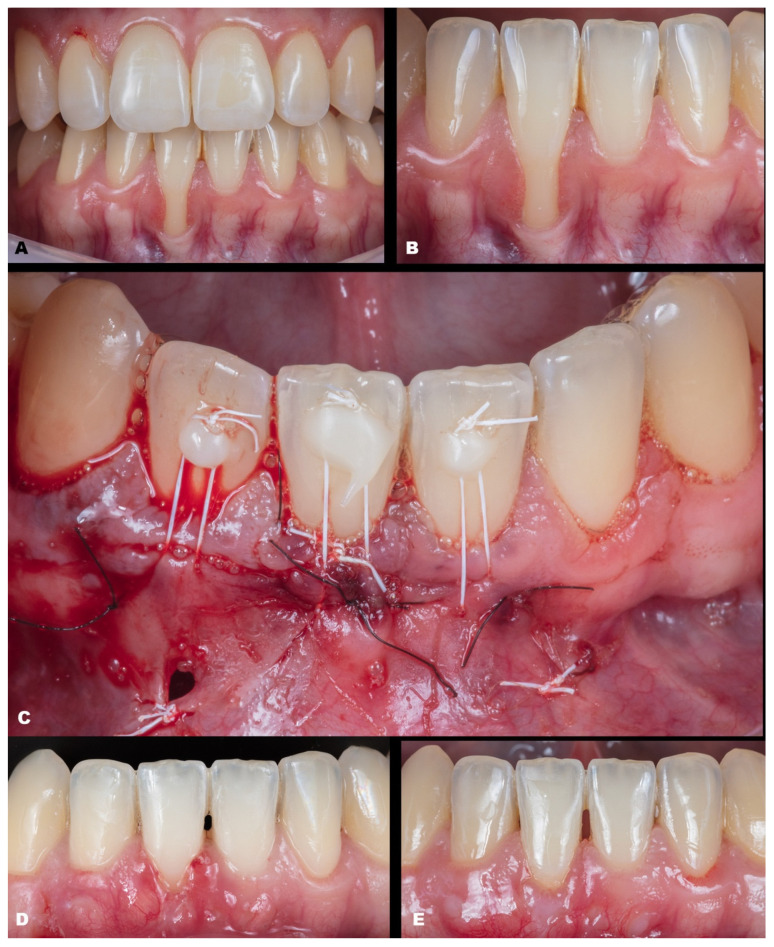
Case 1. (**A**,**B**). Initial pictures of the GR defect (#41). (**C**). Immediately post-operative. (**D**). The healing period of one month. (**E**). The healing after six months.

**Figure 3:**
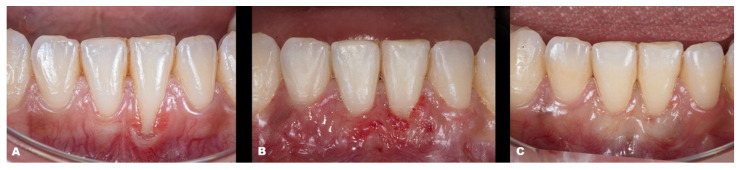
Case 2. (**A**). Initial picture of the GR defect (#31). (**B**). The healing after one month. (**C**). The healing after one year.

**Figure 4:**
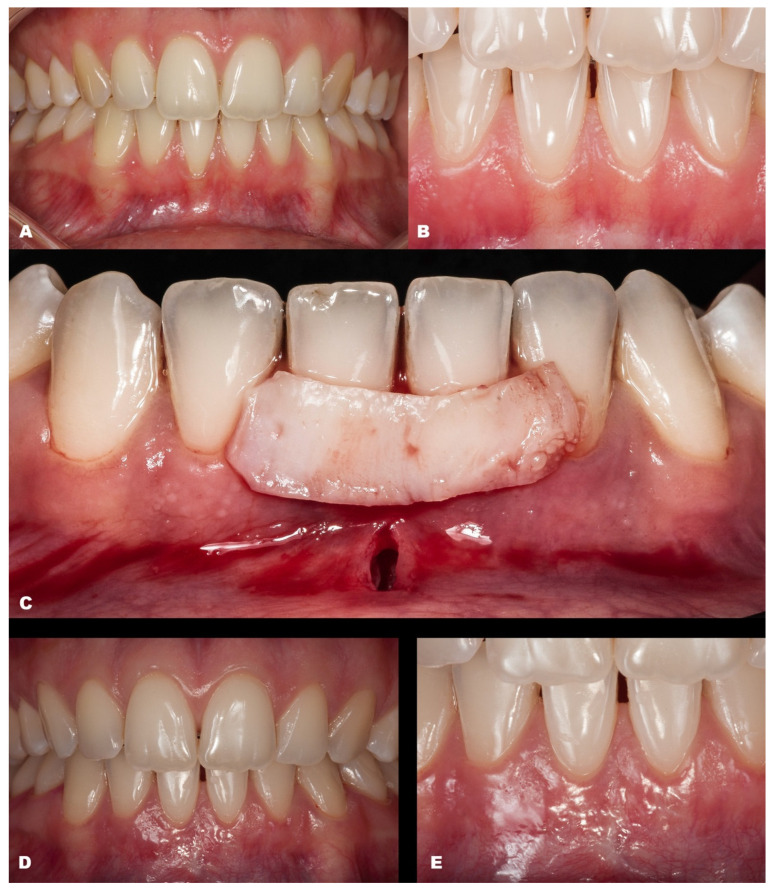
Case 3. (**A**,**B**). Initial pictures of the GR defect (#41) and thin phenotype present. (**C**). CTG over the receptor site and one vertical incision to apply MiTT. (**D**,**E**). The healing after six months.

Case 1 was a male RT2 case involving tooth #41, with slight interproximal bone loss and gingival recession (GR) of 6 mm (height) × 2.5 mm (wide), which achieved the MGJ. No occlusal trauma was detected. The patient reported the use of fixed orthodontic brackets in the past. The MiTT protocol was entirely followed, and the suture was performed with a composite in the buccal face of the teeth for coronal positioning. The result was observed for one year, achieving 82.25% RC (Figure 2). A similar case was performed on a female but involving tooth #31 with 3 mm of GR (RT1), presenting a thin gingival tissue. The patient was scheduled for stitches removal after 7 days and recalled for re-evaluation after 1 month, 6 months, and one year. The result was better than case 1, achieving 100% for RC and improving the tissue thickness (Figure 3). Case 3 involved one tooth (#41–RT1). The recession was 1 mm, and the anterior region had a thin phenotype. In addition, due to the thin thickness of the soft tissue found, the goal was to improve the thickness and cover the root. After applying the MiTT technique with a central vertical incision in the mucosa and using a de-epithelized CTG, an augmented soft tissue thickness was found with a complete RC (100%) (Figure 4).

**Figure 5:**
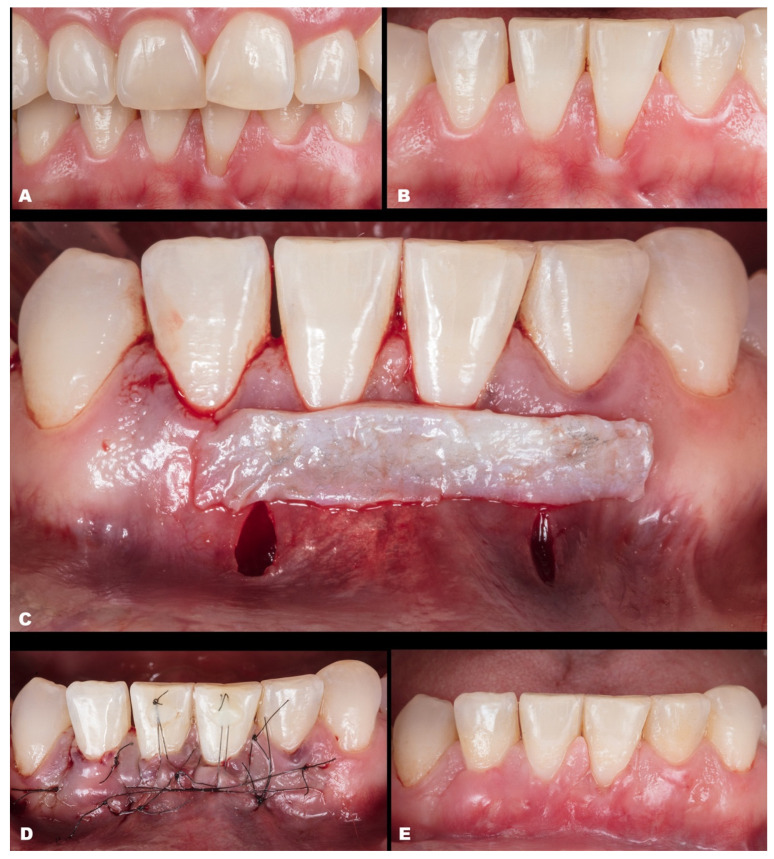
Case 4. (**A**,**B**). Initial pictures of the GR defect (#41 and #31) and thin phenotype present. (**C**). CTG over the receptor site with two distal vertical incisions to apply MiTT. (**D**). Suture performed. (**E**). The healing after six months.

**Figure 6:**
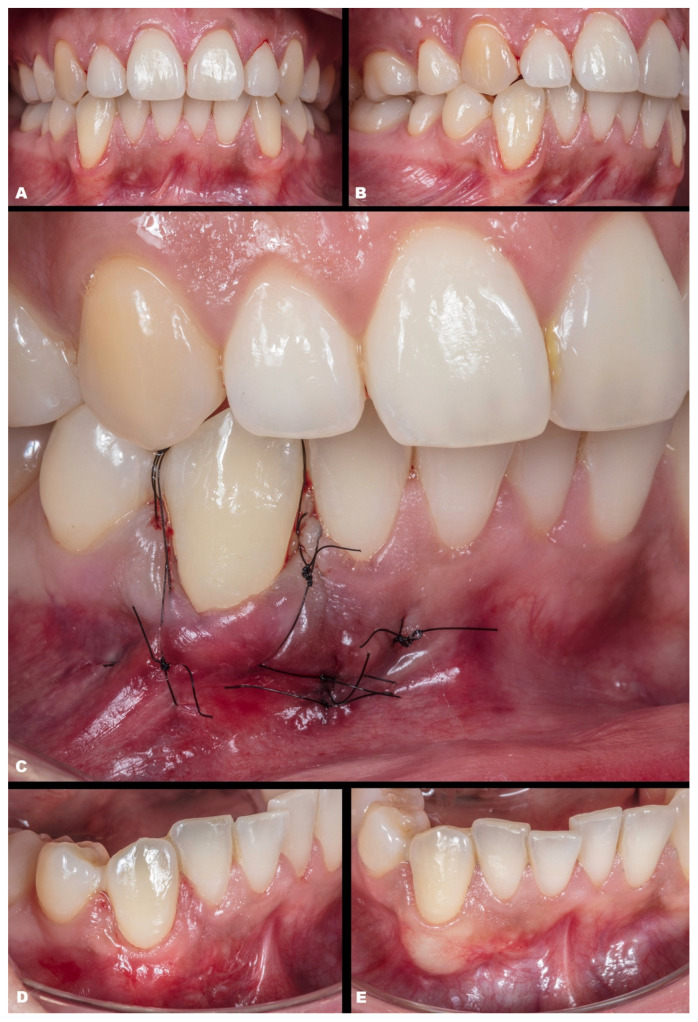
Case 5. (**A**,**B**). Initial pictures of the GR defect (#43). (**C**). Picture of the final aspect immediately after surgery (double-crossed suture). (**D**). The healing period of one month. (**E**). The healing after one year.

**Figure 7:**
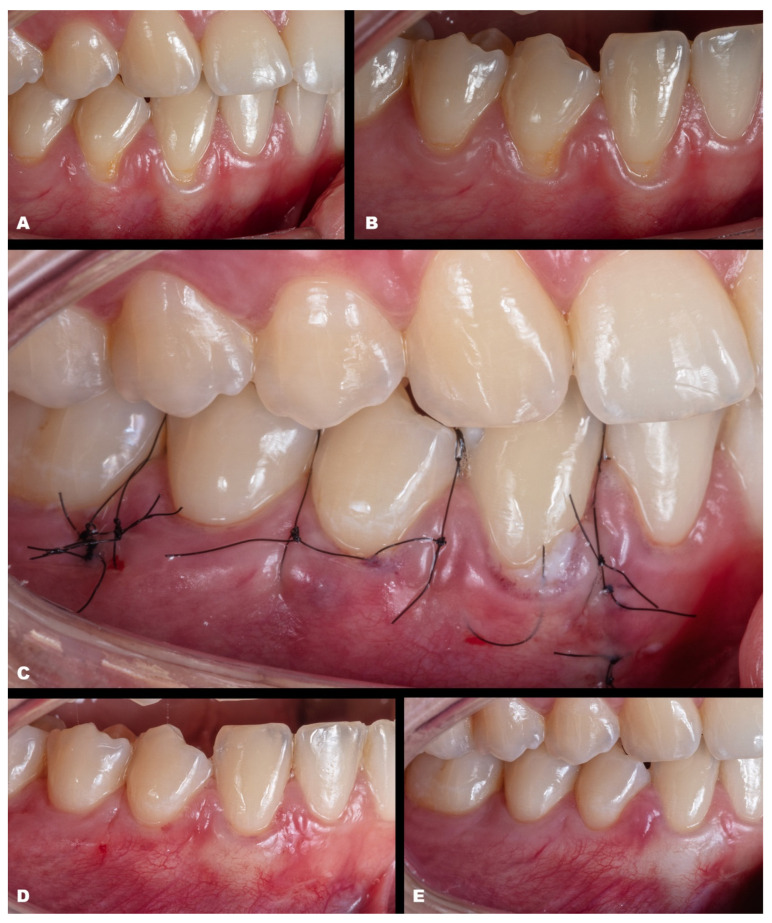
Case 6. (**A**,**B**). Initial pictures of the multiple GR defects (#43–#45). (**C**). The final aspect immediately after surgery (double-crossed suture). (**D**). The healing period of one month. (**E**). The healing after six months.

Case 4 presented GR in teeth #31 and #41, respectively, with 3 mm and 1 mm. The case was approached by applying two vertical incisions instead of one in the distal of each involved tooth. The procedure and tissue manipulation occurred without an event, and the result showed 100% RC (Figure 5). Increasing the difficulty of the tissue management level, case 5 (Figure 6) was performed on a single tooth (#43), which was buccally positioned and classified as RT1, presenting 3 mm of GR. The case was performed in the anterior and buccal regions of the mandible with one vertical incision. After the MiTT technique, a double-crossed suture was made to position the soft tissue coronally and keep it stable. After 7 days and one year, 100% for RC was reached, and a significant tissue volume was found. Another case (Case 6) was also performed in that region but with increased difficulty. Toward the posterior area in the lower arch, three teeth had GR (#43–#45), respectively, with 1 mm, 2 mm, and 1 mm. Rotation was also found in the premolars. The MiTT technique was performed with one vertical incision in the mesial site, and after 7, 14 days, and one year, there was 100% RC with no adverse events, even considering the mental nerve proximity (Figure 7).

**Figure 8:**
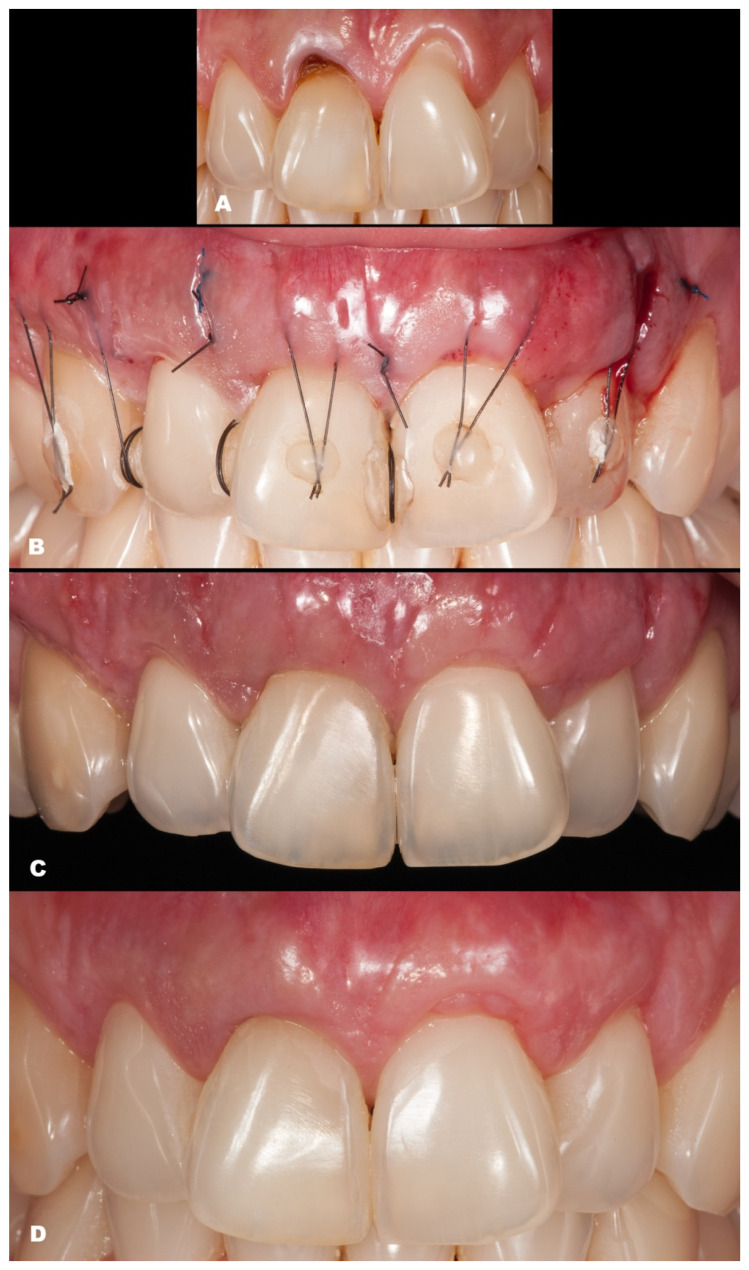
Case 7. (**A**). Initial pictures of the GR defects (#12–#22). (**B**). The final aspect immediately after surgery. (**C**). The healing period of 14 days. (**D**). The healing after one year.

The seventh case (Figure 8) differed from the rest because it involved an esthetic area in an anterior maxillary region. Even with a gingival RT1 in teeth #12–#22, respectively, with 1 mm, 1.5 mm, 2 mm, and 1.5 mm, teeth #11 and #21 presented a wide GR (3 mm and 4 mm, respectively). The case was conducted without adverse events using two linear vertical incisions on the distal to the central incisors. A buccal suture in the facial region of the teeth was made using composite. An adequate healing process was found after 7 days and one year, which was permitted by the tissue stability achieved in soft tissue management. The success rate was 100% for RC.

**Figure 9:**
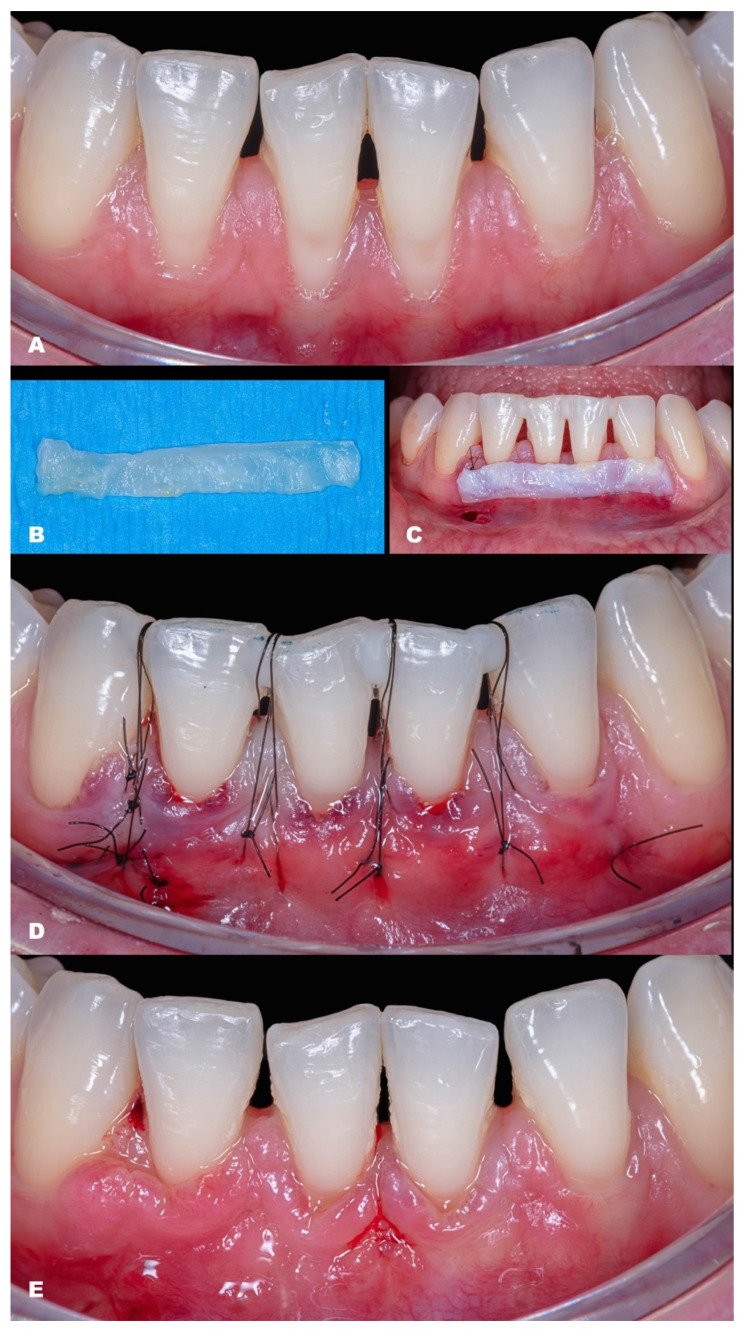
Case 8. (**A**). Initial picture of the multiple GR defects (#42–#32) and thin phenotype present. (**B**,**C**). CTG over the receptor site and two vertical incisions at the distal to apply MiTT. (**D**). The final aspect immediately after surgery. (**E**). The healing period of one month.

The eighth case was an RT2 (Figure 9), with interproximal attachment loss, involving #32 to #42 (GR of 2 mm, 3 mm, 3 mm, and 1 mm, respectively). After explaining that the success rate is reduced in these cases compared to RT1, the patient accepted and was surgically treated. Before starting the procedure, composites were placed in the interproximal of the teeth to apply the double-crossed suture posteriorly [15]. De-epithelized CTG was collected from the hard palate, and two distal vertical linear incisions were made. The mixed tunnel was performed, and the graft was inserted through the tunnel. The suture was coronally positioned. After one year, the healing was favorable, with 100%, 82.35%, 81.25%, and 58.34%, respectively, success rates reached.

**Figure 10:**
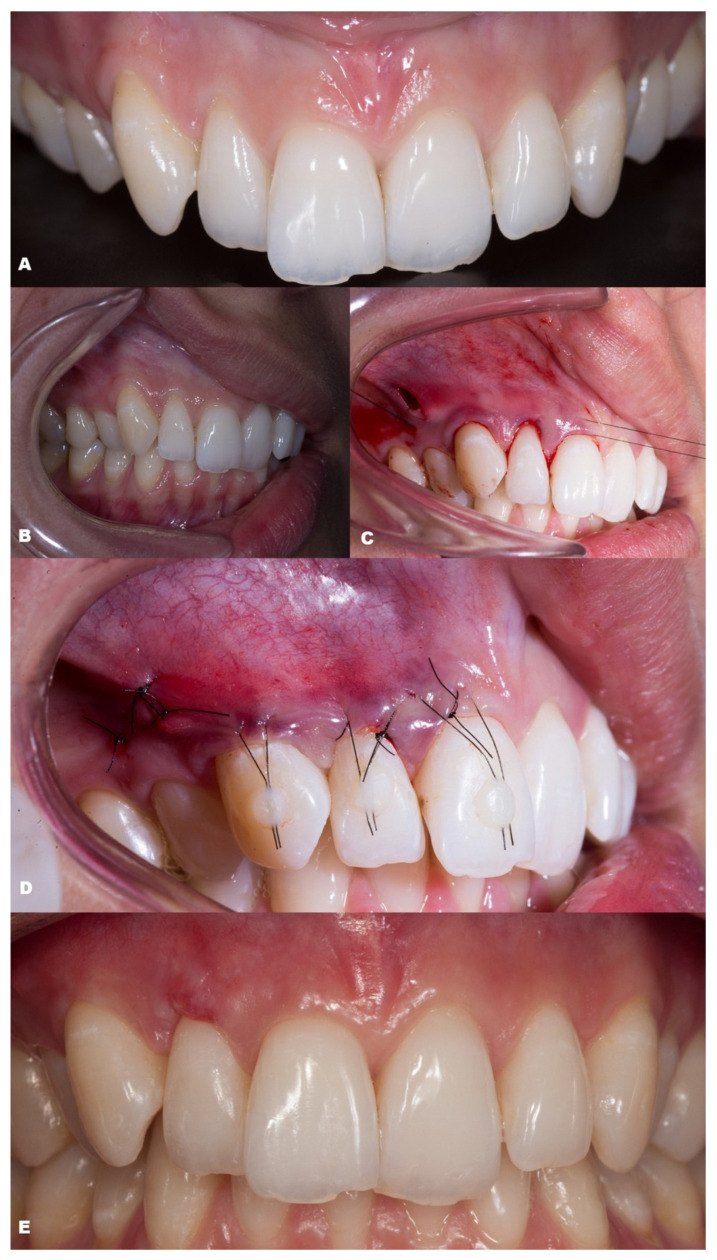
Case 9. (**A**,**B**). Initial pictures of the GR defects (#12–#13). (**C**). CTG positioned through the vertical linear incision. (**D**). Picture of the final aspect immediately after surgery. (**E**). The healing period of six months, with redness at the zenith of tooth #12, local to where the suture was performed.

The last patient (ninth) was an RT1 case involving two adjacent teeth (#11–#12) in the esthetic region (Figure 10). The recession height was 1 mm and 2 mm, respectively. The MiTT surgical steps were followed, and the suture was made with coronal traction of the tunnel, which was held by composites on the buccal face. After 30 days, integration of the CTG was observed with minor dehiscence and redness at the gingival margin. After one year, the healing improved, and complete root coverage was reached.

The healing period was uneventful, with no significant inflammation or direct event related to the MiTT technique, as observed in the patients’ post-operatives. They presented a unanimous report that the discomfort was lower than expected, mainly because of the donor site, and the result was satisfactory after one intervention. Predictably, the RT2 cases had a generally lower percentual success rate compared with the RT1 cases, which obtained complete root coverage for all samples. Also, an increase was found in the keratinized tissue when comparing the baseline and 1-year follow-up. Table 2 shows all data obtained, including the details. Only the recession variable had significant results (*p* > 0.001) after one year. Table 3 shows the advantages, disadvantages, and differences using the MiTT technique.

## 4. Discussion

Gingival recession is the apical migration of the gingival margin, exposing the cementoenamel junction (CEJ) and the root surface [19]. Root coverage procedures aim to treat that defect. Coronally positioned flaps (CAFs) and tunnel techniques (TUNs) have been proposed, associated or not with the CTG [7,20,21,22,23]. Although CAFs, with or without vertical incisions [24,25,26,27], are the most frequently used technique, TUNs have become popular because of the patients’ increasing esthetic awareness and our understanding of the minimally invasive surgery concept [28]. Both are adequate procedures for treating localized and multiple GR defects, with a similar percentage of mean RC, more than 80%, as demonstrated in a systematic study with an extremely high level of heterogeneity [29]. Likewise, a comparison between CAF and TUN using an acellular dermal matrix (ADM) in ≥3 mm Miller class I or II single GR defects showed no statistically significant results [30]. Similarly, contrasting the modified-CAF (MCAF), without vertical incisions [27], to coronally positioned TUN for the treatment of single or multiple Miller class I and II GR defects, both with SCTG [31,32,33], showed an efficient mean RC ranging from 80 to 98%, with no significant results between groups at 6 and 12 months. These findings agree with Toledano-Osorio et al. [34], who reported no differences between techniques, and with González-Febles et al. [35], who evaluated TUN versus CAF in combination with a CTG for the treatment of multiple GRs. These studies found that both groups had similar efficacy in terms of RC; however, TUN demonstrated a higher increase in keratinized tissue (KT), with a milder patient surgical experience. On the other hand, another study showed that TUN resulted in thicker gingiva and better clinical outcomes compared with CAF regarding recession reduction and root coverage [36].

Moreover, new strategies have been developed. One of them is the double-lateral sliding bridge flap technique with a connective tissue graft. It deserves to be cited because it can be applied in sites with a shallow vestibule, high frenum insertion, and/or little or no attached gingiva without any intrasulcular incision [37]. The esthetic result was considered acceptable (final mean esthetic score of 7.4 out of 10), with a mean percentage of RC of 80.5% (mean follow-up of 36 months). As an alternative, this article aimed to introduce the MiTT technique for RC, a tunnel modification using a mixed design (full and split, respectively, subperiosteal and supraperiosteal), which was applied in a case series study. Our mean RC was similar to those found in the literature: 100% for RT1 cases and around 80.83% for RT2 patients.

### 4.1. Evolution of Tunnel Techniques

Historically, to reach the proposed technique (MiTT), the procedure named “envelope” was introduced by Raetzke (1985) [4], which was associated with a subepithelial connective tissue graft (SCTG) into a partial thickness pouch to cover a single recession without any suture. Sequentially, in 1994, the modified envelope technique [6] was introduced to treat multiple recessions with a supraperiosteal approach and some modifications, such as papilla mobilization and graft suturing. It attempted to conserve the existing gingiva and papillae with minimal surgical trauma to the recipient site and keep firm fixation of the connective tissue graft over the areas of recession. Both techniques provided a dual vascular supply (internal and external) to improve graft survival. In 1999, Zabalegui et al. [2] presented the term “tunnel” to describe the preparation of a multi-envelope recipient bed connecting adjacent envelopes, keeping the partial thickness design. Therefore, those techniques did not permit coronal movement of soft tissue, exposing the CTG covering the recessions, which may limit its applicability.

In 2002, Azzi et al. [38] proposed a full-thickness modification in the abovementioned tunnel to permit coronal movement of the entire gingiva–papillary tunnel apparatus. Thus, the advantage was that a large portion of CTG could be covered, avoiding necrosis and achieving a more predictable RC result. This technique was named the modified coronally advanced tunnel (MCAT) technique [39]. Moreover, to minimize trauma, protect the blood supply, and improve wound healing, the tunnel concept was also applied to the microsurgical approach, but still in a supraperiosteal design, using microblades and appropriate instrumentation [40].

Classically, tunnel techniques (TUNs) utilize an incision-free design and preserve the integrity of the papilla, avoiding any detachment, minimizing the risk of losing papilla height and maximizing the blood supply, avoiding scar formation, and keeping an adequate stabilization of the CTG for optimal wound healing [4,28,40]. Otherwise, TUN is a time-consuming and technique-sensitive procedure with limited visibility and access, with the risk of inadequate positioning of the tunnel flap, which can harm the RC outcome. Furthermore, Zuhr et al. [28] suggested that single gingival recession defects exceeding 3 mm in height may be unsuitable for a tunneling approach unless a modification is used. Hence, many surgical changes have been proposed to simplify and improve the technique while retaining elements responsible for the success.

Modifications to the TUN technique have been proposed to improve results and facilitate tissue management. As demonstrated in the introduction section, it is possible to report VISTA [5], PST [8], GDT [9], and M-VISTA techniques [14]. As described, VISTA, PST, and GDT implement periosteal elevation (subperiosteal access), commonly used in dentistry. Even though those techniques present clinical success and preserve the esthetic, two factors might hinder their development/outcome. Firstly, there may be a higher cost of the treatment due to the biomaterials used except for PRF, which has a low cost for preparation. The other is the reduced vascularization for the graft and subperiosteal access, which comes only from the gingiva. Otherwise, no scientific evidence shows any significant questions for the subperiosteal access. However, it is essential to highlight that the recipient flap design contributes to soft graft revascularization and esthetics.

Moreover, the temporal healing sequence at the periosteal–bone interface (PBI) should be considered. After raising the periosteum, the process for new adherence takes at least 12 days for 50% of new insertion and 30 days to obtain 75% of reattachment [40]. In an experimental study [41], the authors demonstrated that control animals (without periosteum elevation or any procedure) had a microscopic normal PBI. After 30 and 90 days of healing, both groups (control and test) had similar results, presenting collagen deposition with minimal cellularity, consistent with an organized scar. As long as a higher number of tissues is raised, a higher inflammatory profile may be found.

On the other hand, M-VISTA [14] implemented a supraperiosteal approach, providing a dual layer of vascularization for the CTG. This technique is highly complex due to the high risk of fenestration, mainly in thin thickness. Thus, this study presented a case series that worked on a new approach (MiTT) to provide a more conservative treatment, following a partial design (apical to the MGJ) and a full design (coronal to the MGJ). It facilitates detachment, improves vascularization of the tunnel, avoids tissue fenestration and scarring, and keeps papilla and esthetic.

Nine patients were included in this study with a 1-year follow-up. Similar data were found by comparing our results (MiTT) to the M-VISTA technique [14]. RT1 cases achieved complete root coverage and a 100% success rate. However, as predicted, RT2-diagnosed patients (2 patients) had lower success rates, with a mean of 80.83%. Moreover, M-VISTA uses a more “aggressive” incision in the labial frenum, which is removed in the MiTT technique. This fact may impair the esthetic result. Compared to MCAT [39], when GR defects approached Miller class I and II, complete RC was found at 42% of test (collagen matrix) sites and 85% of control (CTG) sites, respectively (*p* < 0.05). The mean RC was statistically significant, with 71% at test sites versus 90% at control sites (*p* < 0.05). Values are considered lower than those found in this case series. Also, the mean KTW measured 2.4 ± 0.7 mm at test sites versus 2.7 ± 0.8 mm at control sites (*p* > 0.05). The values were also inferior to those found in this case series after one year, with a gain of 0.9 ± 0.5 mm (final KTW was 4.3 ± 1.4 mm). In addition, unlike the PST and GDT, which make a perforation/hole in the mucosa, the MiTT technique uses a vertical linear incision to preserve local vascularization better [42].

Finally, comparing TUN and CAF techniques associated with CTG allowed significant recession reduction, reaching clinical outcome stability after four years. The TUN had increased KTW and a gain in gingival thickness. However, both techniques were sufficient to keep the patient satisfied regarding esthetic. Otherwise, the esthetic evaluation performed by dentists was more favorable for the TUN technique [43].

### 4.2. Pros and Cons of the MiTT Technique and Limitations of This Study

The Pros of the MiTT technique include (1) vertical incision on the buccal or lingual side, permitting a better vascularization of the tissues; (2) it is not mandatory to perform an intrasulcular incision once the technique uses the vertical incision for detachment, although it might be considered; (3) this technique applies full-detachment design in the keratinized gingiva region, which can be one of the most interesting advantages, avoiding the risk of fenestration in case of thin tissue thickness and keeping a better vascularization; (4) reduced risk of post-operative scarring, preserving the esthetic; (5) no use of flaps, which brings more predictability for the esthetic result; (6) use of a split-detachment design in the region apical to the MGJ improves the tissue mobility, keeping the vascularization; (7) minimal surgical trauma to the recipient area; (8) maintenance of the integrity of the involved papillae, also favoring the esthetic preservation; and (9) the MiTT design permits relative ease suturing while ensuring firm graft fixation and confinement within the recipient site.

The Cons of the MiTT technique include (1) it is necessary to have KTW remnants to obtain a better outcome; (2) the intrasulcular incision can cause some damage in the gingival margin area; (3) tissue detachment through the vertical incision can present some difficulties to be implemented; (4) shortened vestibule may be challenging to perform the procedure; and (5) there is a moderate level of technical sensitivity, which will depend on professional experience/ability.

Regarding this study’s limitations, there was a short-term evaluation even though the tissues were completely stable after one year; it is suggested that future studies apply the long-term assessment. Using MiTT, a reduced vascularization on the bedside was provided for the CTG due to the full-thickness design (subperiosteal) performed, which did not harm the healing or results. The split design (apical to the MGJ) must be carefully completed, observing the thin alveolar mucosa present, avoiding any perforation. This was a case series study testing a new approach for RC with a limited number of patients. The most significant part of the cases was RT1 in the mandible; even though the soft tissue is thinner in this area, increasing the difficulty, more esthetic cases must be performed in the maxilla.

Within the limitation of this case series, the mixed-thickness tunnel access (MiTT) technique can be considered a more straightforward approach for minimally invasive surgical techniques, which is a feasible option to treat RC with a high success rate, predictability, and esthetic preservation. MiTT is a technique-sensitive and time-consuming procedure with limited visibility, which is expected due to the full-split design procedure. Specifically, it can be a treatment option in areas with RT1 and RT2 recessions, with esthetic involvement, shallow vestibules, or a thin phenotype, and in sites with prominent roots (higher risk of laceration). More studies should be developed to evaluate the MiTT technique in the long term and compare it with different approaches.

## Figures and Tables

**Table 1:** Summary of the surgical techniques cited.

	“Envelope” Technique	Modified Envelope Technique	Tunnel Technique	Modified Tunnel Technique	VISTA	PST	m-VISTA	GDT
**Authors/Year**	Raetzke (1985) [4]	Allen (1994) [6,7]	Zabalegui et al. (1999) [2]	Tözüm & Dini (2003) [3]	Zadeh (2011) [5]	Chao (2012) [8]	Lee et al. (2015) [14]	Tuttle et al. (2018) [9]
**Advantages**	- Simple technique (without coronal displacement) with minimal trauma. - Does not detach papillae.	- Can be performed on multiple sites. - Simple technique (without coronal displacement) with minimal trauma.	- Does not detach papillae - Simple technique (without coronal displacement). - Partial dissection.	- Without vertical incisions. - Full-thickness dissection at the mucogingival area (in a coronoapical direction) to supply more blood vessels. - Does not detach papillae.	- Does not detach papillae. - Incision in mucosa facilitating the access. - Easier detachment of the soft tissue (subperiosteal).	- Does not detach papillae. - Incision in mucosa facilitating the access. - Full-thickness dissection (reduced risk of fenestration).	- Double vascular surfaces for revascularization of the graft. - Lower risk of graft necrosis and scarring. - Better capillary ingrowth. - Does not detach papillae.	- Minimally invasive. - Places holes in mucosa to permit the access. - Reduced risk of fenestration-full-thickness tunnel (subperiosteal access). - Does not detach papillae.
**Disadvantages**	- Higher risk of necrosis of the CTG (exposed). - Used in isolated areas (single tooth). - Lower level of root coverage for gingival recessions greater than 3 mm. - Supraperiosteal approach (higher risk of fenestration).	- Supraperiosteal approach—higher risk of fenestration. - Higher risk of necrosis of the CTG (exposed).	- Only intrasulcular incisions. - Supraperiosteal approach (higher risk of fenestration. - CTG has a small exposition—elevated risk of necrosis.	- Only intrasulcular incisions. - CTG has a small exposition (around 50%)—elevated risk of necrosis.	- Expensive biomaterial was used (membrane complex (β-TCP hydrated with rhPDGF-BB)). - More invasive.	- Collagen stripes placed (increase the cost due to the biomaterial). - Specific instruments to perform the technique. - More invasive.	- Access is only through the frenum area, “V-shaped” incision. - Simultaneous frenectomy. - Difficult level for thin phenotypes (risk of fenestration). - More invasive.	- Substitution of the “gold standard” (CTG) for the A-PRF and i-PRF. - Rapid resorption of the PRF compared with the CTG. - More invasive.

A-PRF = advanced-platelet rich fibrin; β-TCP = beta-tricalcium phosphate; CTG = connective tissue graft; GDT = gum drop technique; i-PRF = injectable-PRF; PST = Pinhole^®^ surgical technique; rhPDGF-BB = recombinant human-platelet derived growth factor; VISTA = vestibular incision subperiosteal tunnel access; m-VISTA = vestibular incision supraperiosteal tunnel access.

**Table 2:** Clinical information on the included cases. Data from baseline and after one year.

	Classification	Gender	Tooth/Teeth with REC	Initial REC Height (mm)	Initial REC Width (mm)	Initial PD (mm)	Initial KTW (mm)	Final REC Height (mm)	Final PD (mm)	Final KTW (mm)	% RC	*p*-Value
				**Baseline**				**6-Month Follow-up**				
**Case 1**	RT2	M	41	6.2	2.5	1.0	0.3	1.1	2.0	4.3	82.25	**REC**: *p* < 0.0001 **PD**: *p* = 0.2771 **KTW**: *p* = 0.1013
**Case 2**	RT1	F	31	3.3	1.7	0.5	0.3	none	1.0	3.7	100	
**Case 3**	RT1	F	41	1.2	2.1	1.0	3.3	none	0.5	7.3	100	
**Case 4**	RT1	F	41 31	1.4 1.2	2.1 1.8	1.0 0.5	3.1 2.9	none	1.0 0.5	3.7 4.0	100 100	
**Case 5**	RT1	M	43	1.5	2.3	1.0	1.3	none	1.5	4.4	100	
**Case 6**	RT1	F	43 44 45	1.2 2.1 1.4	2.6 3.1 2.8	2.0 1.5 1.0	3.0 3.8 3.8	none	1.5 1.0 1.0	2.1 2.8 5.3	100 100 100	
**Case 7**	RT1	F	12 11 21 22	1.1 1.5 2.4 1.5	1.5 3.0 4.0 2.0	1.0 2.0 1.5 2.0	5.4 5.0 6.3 6.9	none	1.0 2.5 2.5 1.5	4.3 5.7 6.0 6.3	100 100 100 100	
**Case 8**	RT2	F	42 41 31 32	2.2 3.4 3.2 1.2	2.3 2.5 3.0 2.7	0.5 0.5 0.5 1.0	2.4 1.9 1.5 3.5	0 0.6 0.6 0.5	1.5 1.0 1.0 1.0	3.6 2.9 2.1 3.3	100 82.35 81.25 58.34	
**Case 9**	RT1	F	12 13	1.3 2.1	2.2 2.3	1.5 2.0	4.4 5.3	none	2.5 1.5	4.6 5.2	100 100	

RT = recession type; M = male; F = female; KT = keratinized tissue width; PD = pocket depth; RC = root coverage; REC = recession.

**Table 3:** Advantages, disadvantages, and differences found in MITT.

	MITT
**Advantages**	- Simple technique with reduced risk of fenestration.
	- Does not detach papillae.
	- Easier access.
	- Greater mobility of the tunnel.
	- No exposition of the graft, reduced risk of necrosis.
**Disadvantages**	- Reduced vascularization, complete detachment.
**Differences**	- Lower risk of necrosis for the flap and graft.
	- Used in single or multiple recessions.
	- It can be used in shallow or deep recessions.
	- More predictable release of the tunnel.
	- It is not performed only in the frenum area.

## Data Availability

All data are available in the article.
